# Cyanobacterial removal by a red soil-based flocculant and its effect on zooplankton: an experiment with deep enclosures in a tropical reservoir in China

**DOI:** 10.1007/s11356-018-2572-3

**Published:** 2018-06-26

**Authors:** Liang Peng, Lamei Lei, Lijuan Xiao, Boping Han

**Affiliations:** 1 Institute of Hydrobiology, Jinan University, Guangzhou, 510632 China; 2 Engineering Research Center of Tropical and Subtropical Aquatic Ecological Engineering, Ministry of Education, Guangzhou, 510632 China

**Keywords:** Red soil-based flocculant, Cyanobacteria, Removal, Recovery, Zooplankton, Enclosure

## Abstract

As one kind of cheap, environmentally-friendly and efficient treatment materials for direct control of cyanobacterial blooms, modified clays have been widely concerned. The present study evaluated cyanobaterial removal by a red soil-based flocculant (RSBF) with a large enclosure experiment in a tropical mesotrophic reservoir, in which phytoplankton community was dominated by *Microcystis* spp. and *Anabaena* spp. The flocculant was composed of red soil, chitosan and FeCl_3_. Twelve enclosures were used in the experiment: three replicates for each of one control and three treatments RSBF_15_ (15 mg FeCl_3_ l^−1^), RSBF_25_ (25 mg FeCl_3_ l^−1^), and RSBF_35_ (35 mg FeCl_3_ l^−1^). The results showed that the red soil-based flocculant can significantly remove cyanobacterial biomass and reduce concentrations of nutrients including total nitrogen, nitrate, ammonia, total phosphorus, and orthophosphate. Biomass of *Microcystis* spp. and *Anabaena* spp. was reduced more efficiently (95%) than other filamentous cyanobacteria (50%). In the RSBF_15_ treatment, phytoplankton biomass recovered to the level of the control group after 12 days and cyanobacteria quickly dominated. Phytoplankton biomass in the RSBF_25_ treatment also recovered after 12 days, but green algae co-dominated with cyanobacteria. A much later recovery of phytoplankton until the day of 28 was observed under RSBF_35_ treatment, and cyanobacteria did no longer dominate the phytoplankton community. The application of red soil-based flocculant greatly reduces zooplankton, especially rotifers, however, Copepods and Cladocera recovered fast. Generally, the red soil-based flocculant can be effective for urgent treatments at local scales in cyanobacteria dominating systems.

## Introduction

Cyanobacterial blooms with eutrophication are increasing throughout the world because of deteriorating global environment (Codd 2000; de Figueiredo et al. 2004; O’Neil et al. 2012; Jeppesen et al. 2017). Cyanobacterial blooms and cyanotoxins pose high risks to human health and have triggered a series of problems for aquatic ecosystems through such processes as diminishing dissolved oxygen, decreasing water transparency, reducing biodiversity, and producing toxins and off-odors (Lindholm et al. 1989; van Apeldoorn et al. 2007; Dash et al. 2015). Not surprisingly, controlling cyanobacterial blooms have become one of the leading issues for ecosystem management (Hrudey et al. 1999; Paerl et al. 2011). Negative impacts of cyanobacterial blooms on aquatic ecosystems have led to suggestions of an upper limit of algal cells in the management of lakes and reservoirs, especially those for drinking water supply (Qin et al. 2010; Newcombe et al. 2010). The World Health Organization also proposed an alert level for drinking water (Chorus and Bartman 1999).

Direct control measures of cyanobacterial biomass are usually grouped into three categories: (1) physical materials such as UV light, ultrasonication, and filtration (Lawton et al. 1998; Rajasekhar et al. 2012); (2) chemical coagulation, flocculation, and algicide (Jeune et al. 2006; Teixeira and Rosa 2006); and (3) biological inhibition and degradation using microorganisms, grazers, and extractions from organisms (Hunt and Matveev 2005; Ji et al. 2009; Combes et al. 2013). Flocculants combined with various substances have been widely used as an efficient, rapid and low cost method of controlling cyanobacterial density in natural water (Sridhar et al. 1988; Sengco et al. 2001; Beaulieu et al. 2005; Lee et al. 2008). Materials such as metal ions, plant seeds, and synthetic organic matter are able to significantly improve the efficiency of flocculants in cyanobacteria removal (Chow et al. 1998; Robb et al. 2003; Liu et al. 2010; Teixeira et al. 2010; Nishi et al. 2012). Clays have different removal efficiency, which depending on cyanobacterial species and phytoplankton communities (Avnimelech et al. 1982; Pan et al. 2006; Verspagen et al. 2006; Anderson 2009; Lürling and Faassen 2012; Vandamme et al. 2013). Effects of flocculation on aquatic animals and macrophyte in water bodies have been investigated (Lewis et al. 2003; Akeprathumchai et al. 2004; Archambault et al. 2004; Lee et al. 2008; Seo et al. 2008). Some modified clays show low toxicity to cladocera and copepods, while Lanthanum addition was found to be chronically toxic to *Daphnia* spp. (Stauber 2000). The EC_50_ (half maximal effective concentration) of a modified clay on the population growth of rotifer is suggested by van Oosterhout and Lürling (2013) to be suitable for field application. However, low concentration clay has less effect on *Daphnia*, although the animals became smaller, matured later, and reproduced less as lanthanum increases, resulting in lower population growth rates in the presence of phosphate (Lürling and Tolman 2010). Clay flocculation is thought to be helpful for the restoration of submerged macrophyte with improved water quality (Boustany 2003).

In China, cyanobacterial blooms caused by *Microcystis*, *Anabaena*, *Aphanizomenon*, and *Cylindrospermopsis* are a common occurrence in many lakes and reservoirs (Qin 2002; Cai et al. 2012; Lei et al. 2012). The blooms are mainly observed in the dry season from late autumn to spring in southern China, a phenomenon quite different from that in the temperate zone. The absence of cyanobacterial blooms in the wet (monsoonal) season is usually attributed to high flush rate, which strongly reduces phytoplankton density. However, high amounts of suspended inorganic particles washed from the red soil watersheds may also play an important role in flocculation. Wang et al. (2012) found a significant negative relationship between particle concentration and phytoplankton density in a large canyon reservoir. Flushing water with clay is effective at mitigating cyanobacterial growth and at suppressing cyanobacterial blooms (Mitrovic et al. 2011). Red soils are rich in iron in southern China (Xu et al. 2006) and contribute suspended inorganic particles in watersheds, which appear effective in flocculation. Xiao et al. (2007) and Liu (2016) reported modified red soil with iron is more efficient reducing cyanobacterial biomass than natural red soil. In practical application, we add chitosan as an auxiliary coagulant to enhance flocculation as early suggestion (e.g., Pan et al. 2011). However, the removal capacity of such new modified red soil (red soil-based flocculant, RSBF) has not been quantitatively evaluated, and there is a little information regarding the possible effect on other phytoplankton species and zooplankton. In the present study, we applied one of the new modified red soils in a large enclosure experimental system to test (1) the removal efficiency of different cyanobacterial species and its influence on phytoplankton community, (2) the recovery time of cyanobacteria, and (3) the possible negative impact on zooplankton community.

## Materials and methods

**Fig. 1:**
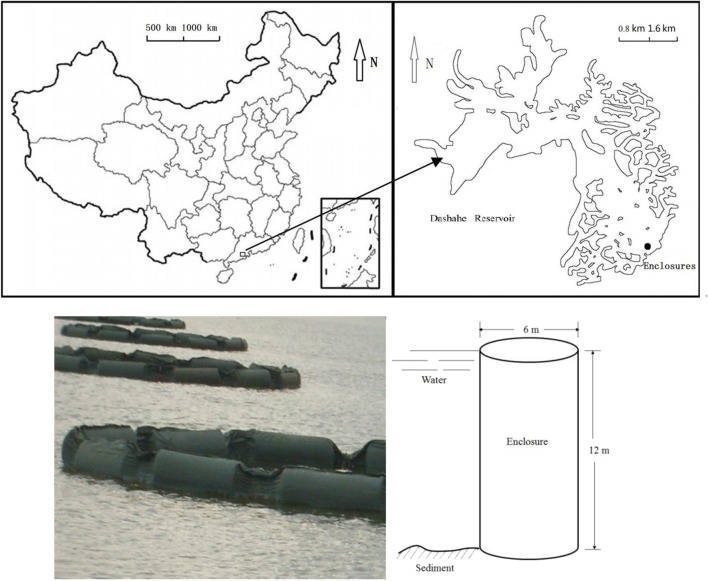
Location and diagram of enclosures in the studied reservoir: Dashahe reservoir, southern China

Twelve enclosures were installed in the tropical mesotrophic Dashahe Reservoir (N 22.52′, E 112.42′) in southern China (Fig. 1). During the experimental period, water temperature ranged from 28.4 to 30.4 °C, pH value ranged from 6.2 to 7.8, turbidity ranged from 241.2 to 335.4 NTU, and salinity was stable at 0.04. Each enclosure has a volume of 340 m^3^ (6 m diameter, 12 m deep). The enclosures were made of polypropylene, and were open at the top and bottom. The upper mouth was framed with a steel rim fixed with a floating system, which kept the top of enclosure out of the water (0.5 m above the water surface) and the enclosures in upright position. The end rim of each enclosure was inserted into the sediment by a weighed stone line. There was no water exchange between the enclosures and the surrounding water except that from the sediment.

Ferric trichloride and chitosan of analytical pure were the production from Sinopharm Group CO.LTD (China). Local red soil, ferric trichloride, and chitosan were screened with a 0.85-mm mesh sieve and mixed together. There were three experimental treatments each with a different combination of concentrations of RSBF (termed RSBF_15_, RSBF_25_, and RSBF_35_ Table 1). Each treatment plus the control have three replicates. Water samples were collected from each enclosure every 4 days for nutrient measurement and phytoplankton and zooplankton species counts. The RSBF was added only once into the nine treatment enclosures just after the first sampling. The experiment started on August 25, 2008 and ended on October 4, 2008.

**Table 1:** Final concentration of the red soil-based flocculant (RSBF) used in the experiment

	FeCl_3_ (mg l^−1^)	Chitosan (mg l^−1^)	Local red soil (g l^−1^)
Control	0	0	0
RSBF_15_	15	2.5	0.13
RSBF_25_	25	4.2	0.17
RSBF_35_	35	5.8	0.23

Temperature and pH were measured with a portable YSI at a depth of 0.5 m below the water surface. Water transparency in Secchi depth (SD) was measured with Secchi disk. Nutrient concentrations were measured following the national standard methods published by Chinese EPA (China National Standards 1987a, b, 1989a, b). Chlorophyll *a* was extracted with 90% acetone and then determined with a spectrophotometric method (Lin et al. 2005). Phytoplankton was collected from the surface to 0.5 m depth of the water column and preserved with formalin solution (0.5%). Phytoplankton samples were identified under inverted microscopy using Utermöhl chambers according to Lund et al. (1958) to the species level. Biovolume of each species was calculated based on the morphology of cells according to Hillebrand et al. (1999). Zooplankton was sampled with a 5-L sampler at 1-m interval from a depth of 0.5 to 12 m (near the bottom). Zooplankton was gathered and filtered with a plankton net (mesh size 38 μm), then stored in 5% formalin solution. Zooplankton was sampled three times during the experimental period—the first day, day 10, and day 20, and identified to species and counted for abundance under the microscope (Koste 1978; Shen and Song 1979; Korovchinsky 1992).

For three treatments and the control, the comparison between treatments was analyzed by one-way ANOVA. Two-way mixed repeated measure ANOVA in generalized linear model was also used for time series observation. We first detected the variances of the differences between all combinations of groups of within-subjects factors by the Mauchly’s tests of sphericity. If there is no homogeneity of variances, the correction by adjustment of freedom degrees was applied. The treatments were compared by post hoc tests. Bonferroni correction was used to compare main effects. As flocculant was added only once, the phytoplankton and zooplankton started to recover 1 week later, more groups of within–subjects will reduce the difference between the time and the treatment. Thus, we used both ANOVA and repeated measure ANOVA to detect the effect of the flocculant in the treatments. An SPSS statistical package (release 11.5 for Windows) was used for all statistical analysis.

## Results

### Effects of RSBF on water quality

**Fig. 2:**
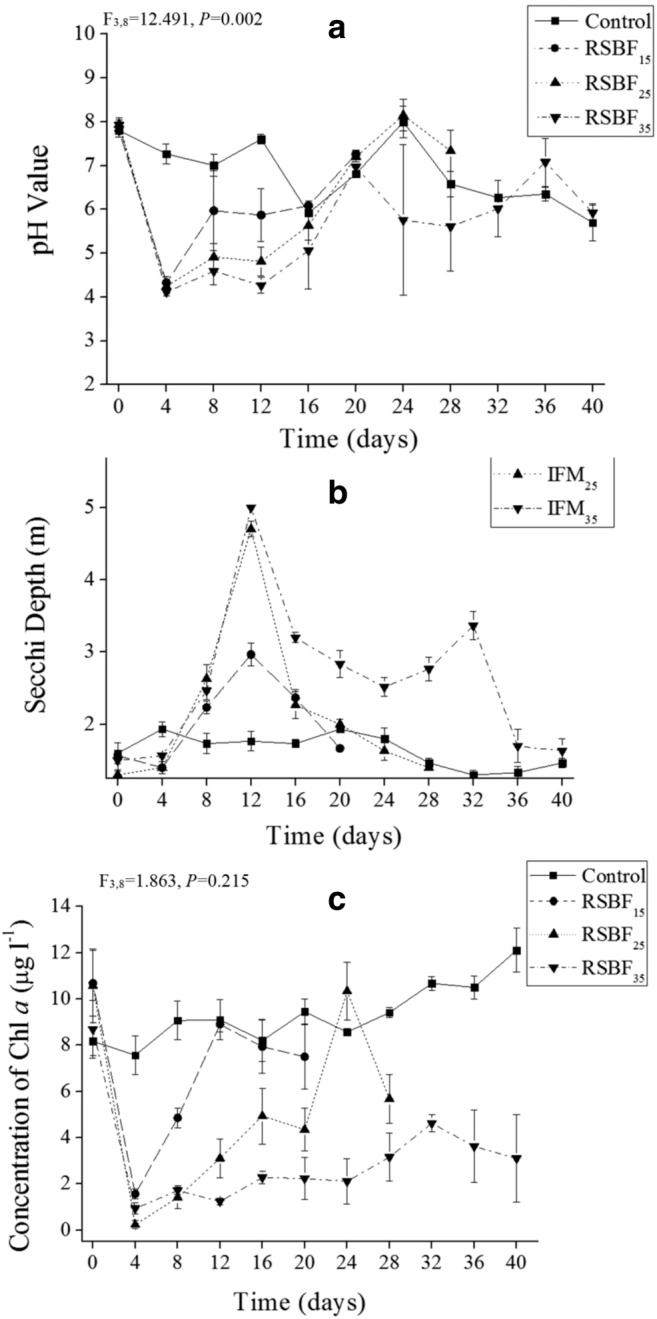
Dynamics of pH value (**a**), SD (**b**), and concentration of chlorophyll *a* (**c**) in three treatments. Error bar indicates standard deviation

Addition of the flocculant mixture rapidly decreased the pH value (Fig. 2a), which reached a low of about 4.1 in all three treatments. A gradually increasing pH value was then observed until day 20, reaching pH of 5.8 in the RSBF_35_ treatment, 6.2 in the RSBF_25_, and 7.1 in the RSBF_15_ treatments. The pH value in the control enclosures was about 7.0, which was significantly higher than those in the RSBF_35_ treatment (*p* < 0.05, ANOVA).

Secchi depth in the control was below 2 m throughout the experiment (Fig. 2b). However, an increased water transparency was observed in the RSBF treatments from the eighth day, peaking at 5 m on day 12. While high water transparency continued until day 32 in the RSBF_35_ treatment.

Chlorophyll *a* declined rapidly in the first 4 days in the RSBF treatments (Fig. 2c), but it recovered later. Chlorophyll *a* concentration in the RSBF_15_ treatment recovered to a level similar to that in the control after 12 days. It took RSBF_25_ 24 days to reach the same level as that in the control. The Chlorophyll *a* concentration in the RSBF_35_ treatment always remained at a relatively low level until the end of the experiment.

**Fig. 3:**
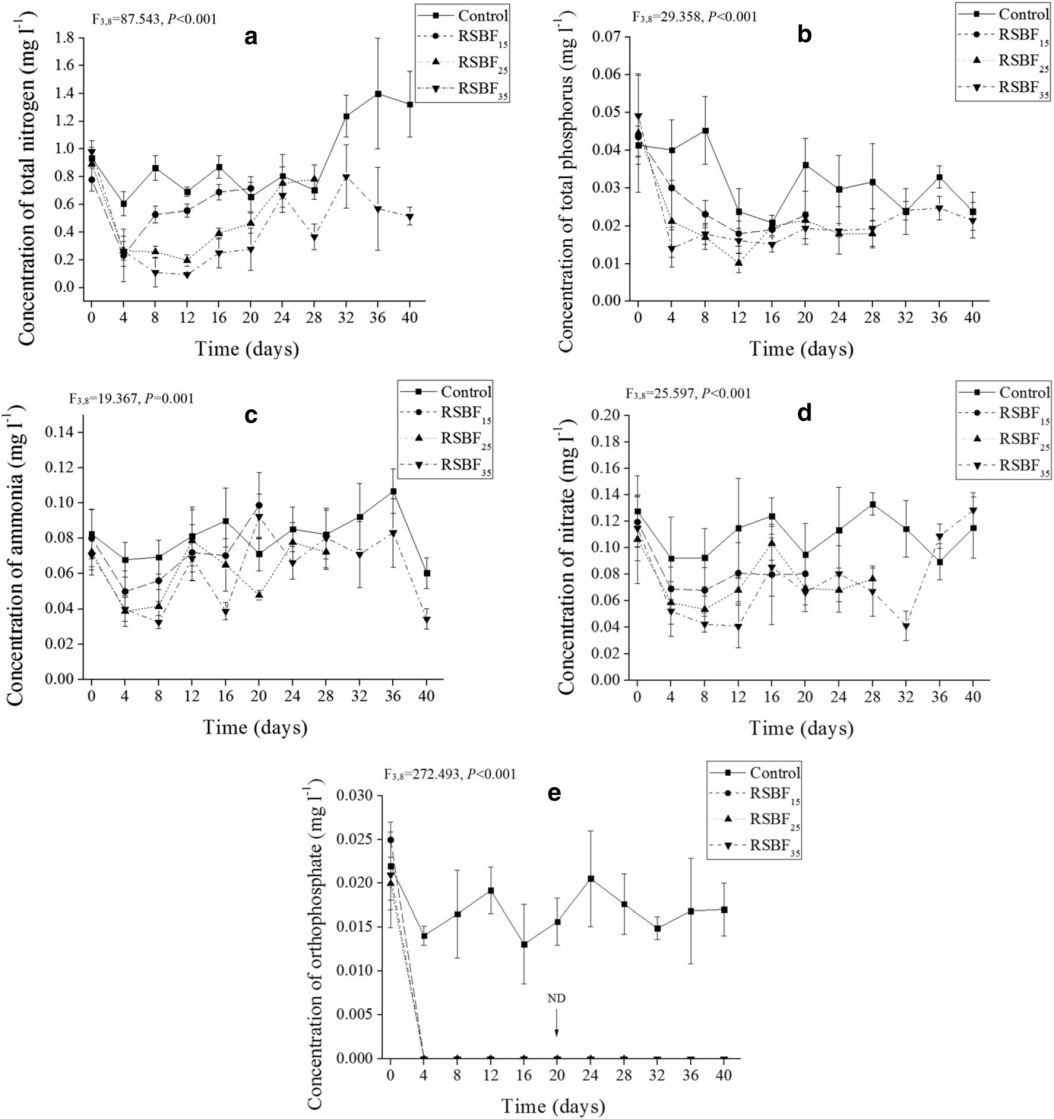
Dynamics of nutrients concentration. **a** Total nitrogen. **b** Total phosphorus. **c** Ammonia. **d** Nitrate. **e** Orthophosphate. Error bar indicates standard deviation

Total nitrogen (TN), nitrate, ammonia, total phosphorus (TP), and soluble reactive phosphorus (SRP) declined significantly in the three treatments in the first week after RSBF was added compared with initial values (Fig. 3). Concentration of SRP was too low to be detected out by our instrument.

### Removal of phytoplankton and cyanobacteria

At the beginning of the experiment, about 30 phytoplankton species were identified from all enclosures; these belonged to cyanobacteria, Chlorophyta, Bacillariophyta, Pyrrophyta, and Euglenophyta. Cyanobacterial species such as *Microcystis* spp. and *Anabaena* spp. predominated in the phytoplankton community, followed by filamentous *Cylindrospermopsis* spp*.*, *Limnothrix* spp*.*, and some Chlorophyta species, including *Staurastrum gracile*, *Staurastrum dejectum*, *Tetraedron minimum*, *Scenedesmus quadricauda*, and *Quadrigula chodatii.*

**Fig. 4:**
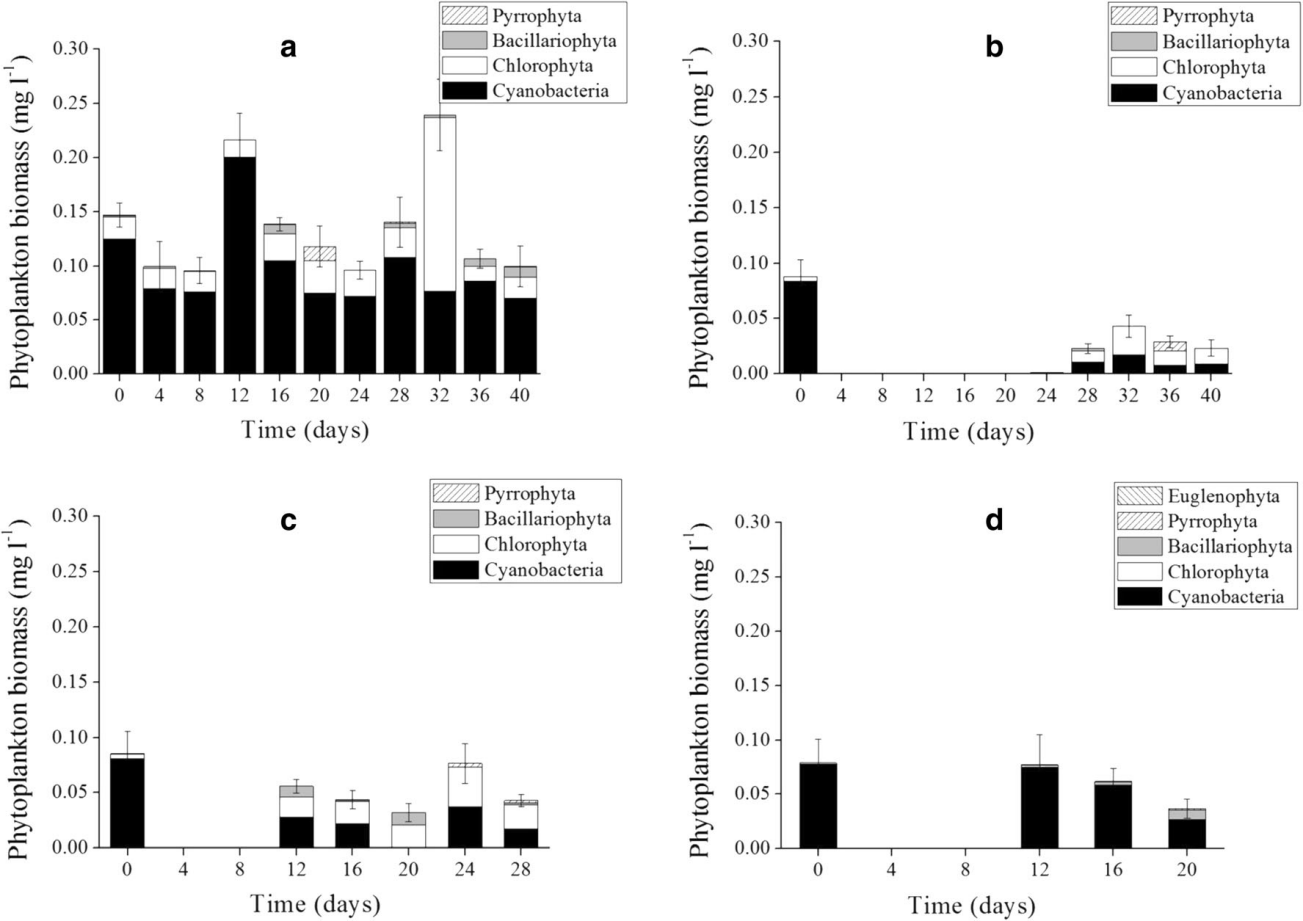
Variation in phytoplankton biomass under four concentrations of RSBF. **a** Control. RSBF_35_ (**b**). RSBF_25_ (**c**). RSBF_15_ (**d**). Error bar indicates standard deviation

**Fig. 5:**
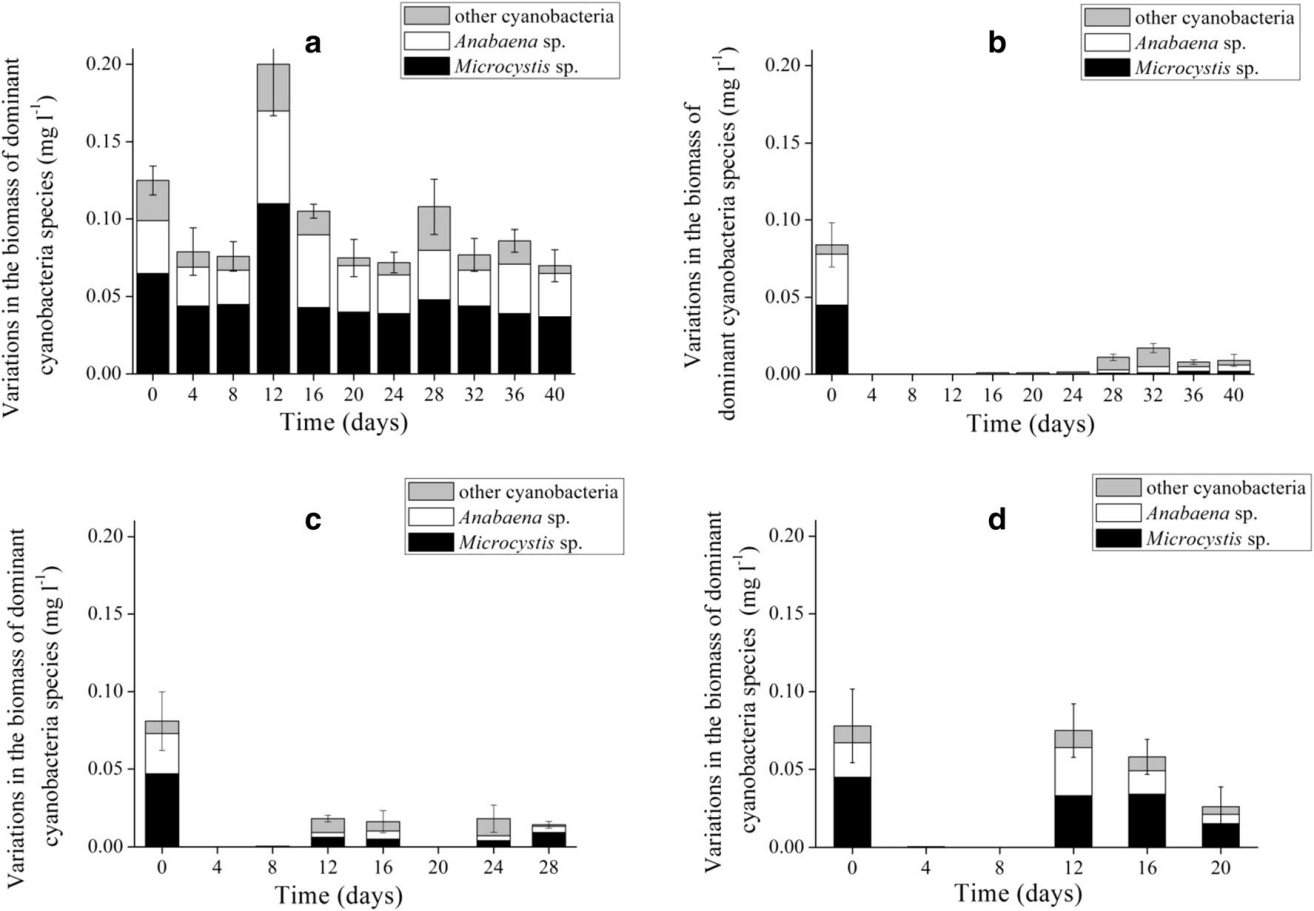
Variation in the biomass of dominant species under four concentrations of RSBF. **a** Control. Treated with RSBF_35_ (**b**), RSBF_25_ (**c**), and RSBF_15_ (**d**). Error bar indicates standard deviation

Cyanobacteria biomass in the control enclosures contributed to more than 70% of the total phytoplankton biomass except on the day 32, when *Cosmarium* spp. (Chlorophyta) dominated (Fig. 4a). *Microcystis* spp. and *Anabaena* spp. were dominant in the controls throughout the experimental period, while filamentous species such as *Pseudoanabaena* spp*.*, *Cylindrospermopsis* spp., and *Limnothrix* spp. comprised only a very small proportion (Fig. 5a).

Biomass of *Microcystis* spp., *Anabaena flos-aquae*, and other filamentous species declined markedly in the three treatments in the first 12 days after RSBF was added (*p* < 0.05, ANOVA. Fig. 5). In the RSBF_35_ treatment, phytoplankton biomass was almost completely removed, and only few algal cells were found, until day 28. More than 95% of cyanobacteria were removed in the experimental period (Fig. 4b). Towards the end of the experiment, cyanobacteria and Chlorophyta began to predominate, followed by diatoms. Chlorophyta biomass increased faster than others, and contributed more than 50% of the total biomass. Dominance of cyanobacteria was below 30%, although phytoplankton biomass remained low (Fig. 4b). Average removal rate of *Microcystis* spp., *Anabaena* spp., and other filamentous species was 99, 96, and 52%, respectively.

More than 80% of cyanobacteria was removed in the RSBF_25_ treatment (Fig. 4c). Phytoplankton biomass recovered on day 12, when cyanobacteria, Chlorophyta, and Bacillariophyta co-dominated (Fig. 4c). The dominance of cyanobacteria was less than 40%. Average removal rate of *Microcystis* spp., *Anabaena* spp. and other filamentous species was 91, 90, and 47%, respectively.

In the RSBF_15_ treatment, removal rate for cyanobacteria was about 40% (Fig. 4d). From day 12 until the end of the experiment, cyanobacteria exclusively dominated the phytoplankton assemblage again (Fig. 4d), with *Microcystis* spp. and *Anabaena* spp. having recovered quickly at the end of period. The removal rate was 67% for *Microcystis*, 55% for *Anabaena* and 43% for the other filamentous cyanobacteria.

**Fig. 6:**
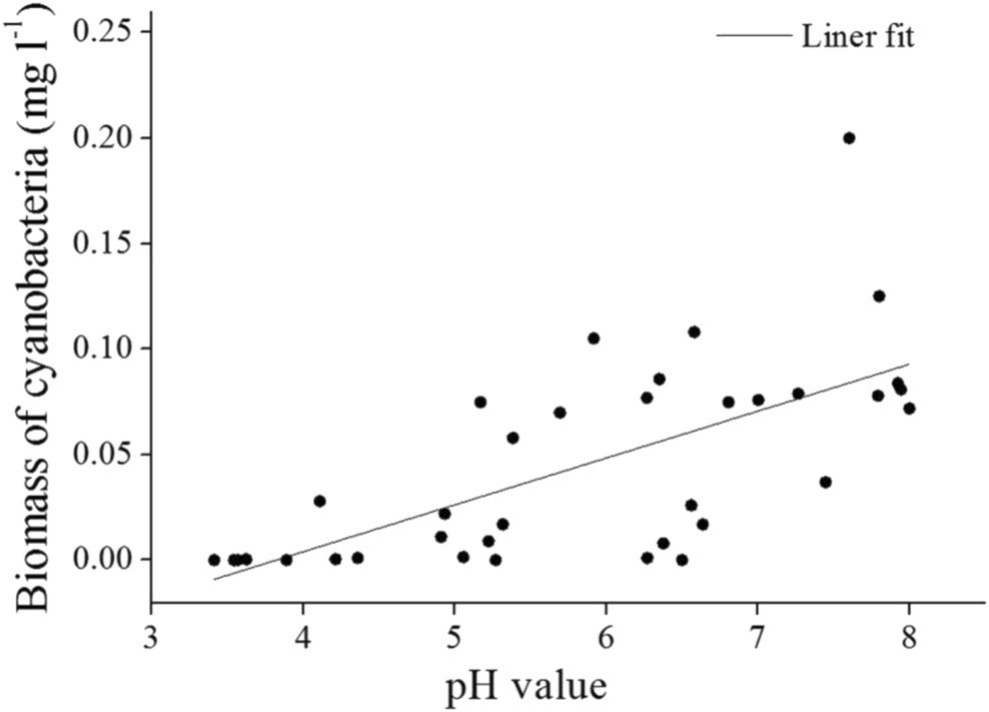
Relationship between pH value and biomass of cyanobacteria: Biomass = 0.0223 × pH–0.085

There was a linear relationship between pH value and biomass of cyanobacteria (*R*^2^ = 0.45, *p* < 0.001; Fig. 6), with the equation: Biomass = 0.0223 × pH–0.085. Variation in the percent of cyanobacteria biomass in phytoplankton was independent of pH (*R*^2^ = 8 × 10^−6^; *p* > 0.05).

### Effects of RSBF on zooplankton community

About 22 zooplankton species were identified in all enclosures. The most common rotifers species were *Keratella cochlearis*, *Brachionus forficula*, *Polyarthra vulgaris*, *Trichocerca cylindrica*, and *Filinia opoliensis*. For Cladocera, *Bosmina longirostris* and *Bosminopsis deitersi* were the common species. The Copepoda were dominated by *Thermocyclops taihokuensis*, *Heliodiaptomus serratus* and their copepodites, and nauplii.

**Fig. 7:**
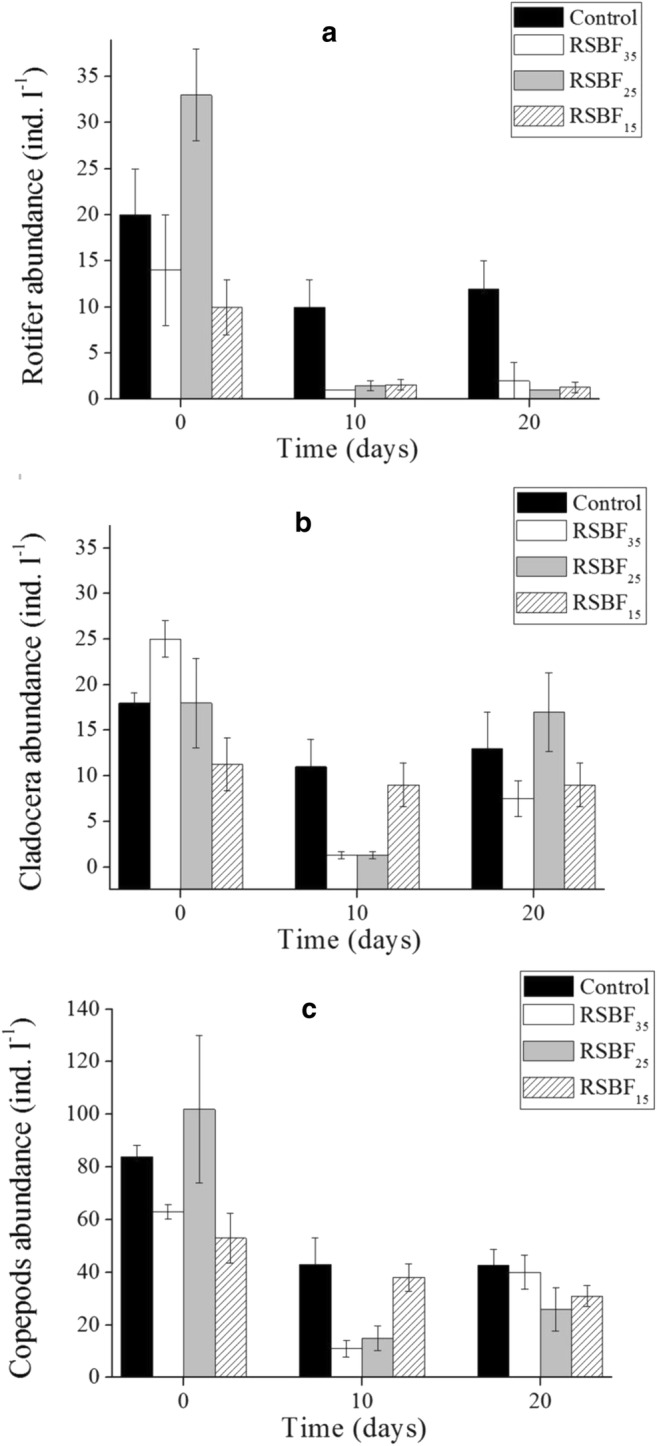
Dynamics of zooplankton abundance for Rotifera (**a**), Cladocera (**b**), and Copepoda (**c**). Error bar indicates standard deviation

Copepods dominated the zooplankton assemblage in all experimental enclosures at the beginning of the experiment. Rotifer density in the three treatments quickly declined to less than two individuals per liter (Fig. 7a). Most rotier species disappeared by day 20, including *Anuraeopsis fissa*, *Brachionus angularis*, *B. calyciflorus*, *Keratella tropica*, *Trichocerca stylata*, *T. rousseleti*, *T. pusilla*, *Collothecidae pelagica*, and *Hexarthridae mira*. Recovery of rotifers in the treatments was very slow.

**Fig. 8:**
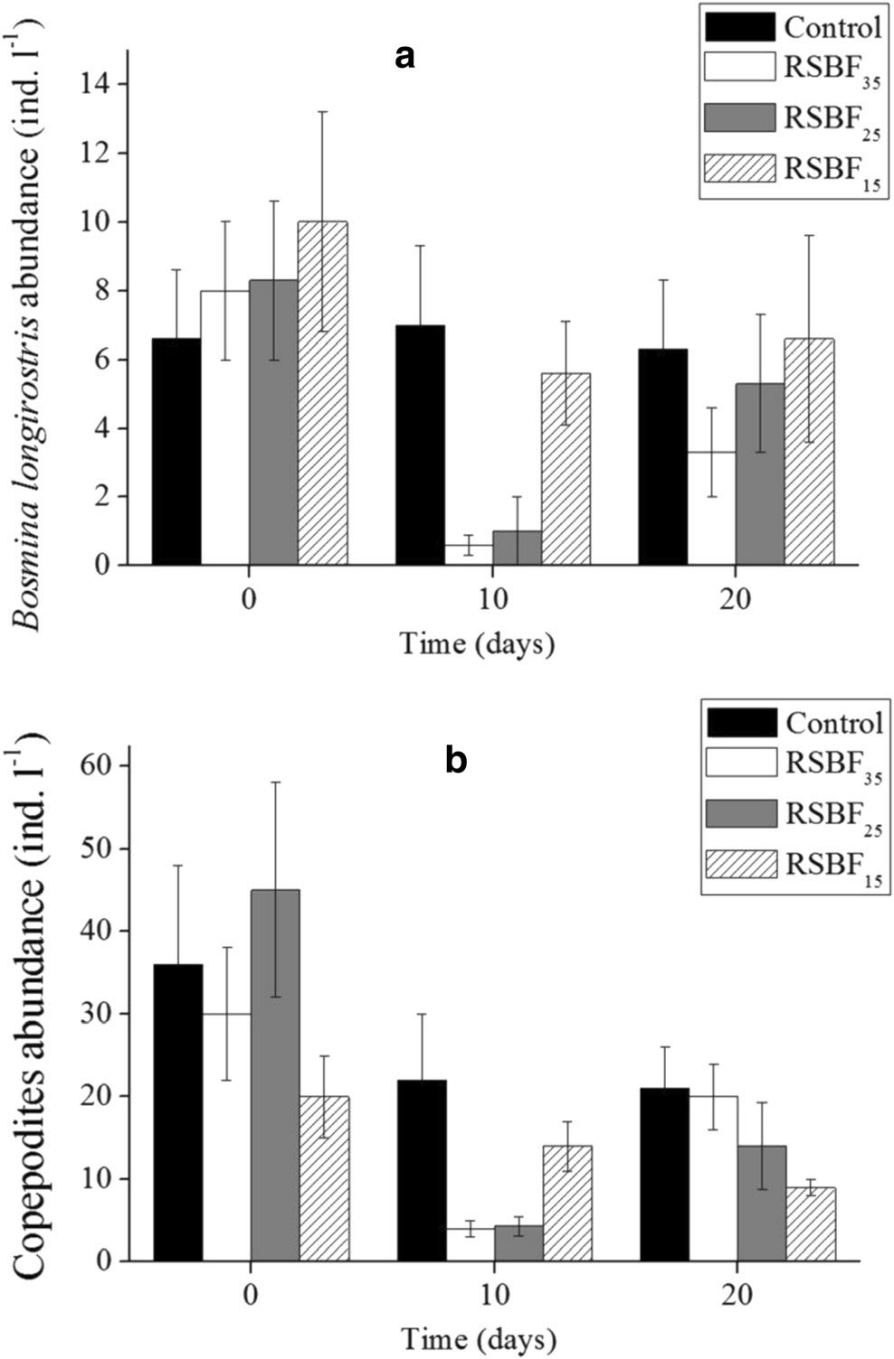
Dynamics of zooplankton abundance for *Bosmina longirostris* (**a**) and Copepodites (**b**). Error bar indicates standard deviation

Cladocera, which were dominated by *Bosmina longirostris* and *Bosminopsis deitersi* Richard, was significantly affected by RSBF_25_ and RSBF_35_ (Fig. 8a). Although *Thermocyclops taihokuensis* was the dominant copepod species, the community biomass comprised mainly copepodites and nauplii (> 60%, Fig. 8b). The effect of the flocculant mixture on Copepoda was similar to that on Cladocera. Copepods and Cladocera recovered faster than Rotifers under RSBF treatments, but species composition remained unchanged (Fig. 7).

## Discussion

### Removal of cyanobacteria by RSBF

Our experiment indicated that RSFB_25_ and RSFB_35_ can effectively remove *Microcystis* spp. and *Anabaena* spp. Flocculation of similar clays has been practiced for mitigating harmful algal blooms (Sengco and Anderson 2004). For example, using modified clay in Lake Taihu in China, Pan et al. (2011) showed that 99% of the cells in a cyanobacterial bloom were removed after only 16 h. The present study showed that RSBF_25_ and RSBF_35_ are capable of removing more than 95% of cyanobacteria within 4 days, especially for *Microcystis* spp. and *Anabaena* spp., which are the causing species for most of the blooms in China.

Teixeira et al. (2010) reported that the main coagulation mechanism of flocculating cyanobacterial cells by clay was due to charge neutralization. In the presence of an electrolyte, algal cells form aggregates with clay particles (Avnimelech et al. 1982). Both size and fractal dimension of flocculants were the most important parameters influencing removal efficiency (Han and Kim 2001). Stickiness on the surface of some cyanobacterial cells showed a significant positive correlation with the amount of extracellular uronic acids, which would play an important role in aggregation through the formation of cation bridges (Verspagen et al. 2006). Our results showed that RSBF is more efficient for *Microcystis* spp. and *Anabaena* spp. removal than for the other filamentous species such as *Cylindrospermopsis* spp. and *Limnothrix* spp. Both *Microcystis* spp. and *Anabaena* spp. secrete a layer of mucilage that could be useful in algal aggregation. Moreover, spherical cells of these two species could be bound more easily by the flocculants than do filamentous species. Chitosan is a linear polysaccharide composed of randomly distributed β-(1-4)-linked D-glucosamine and N-acetyl-D-glucosamine, and has the ability to bridge between individual flocs. Addition of chitosan may improve flocculation (Lertsutthiwong et al. 2009).

Flocculants such as ferric chloride are considered friendly flocculants that neither damage the structure of cyanobacterial cells nor cause acute release of cyanotoxins from the cells (Chow et al. 1998). Peterson et al. (1995) found that cell membrane of *Aphanizomenon flos-aquae* could not lysis under 25 mg l^−1^ of FeCl_3_. Furthermore, ferrate could remove cyanobacterial peptide toxins effectively from eutrophic waters (Yuan et al. 2002). In our experiment, cyanobacterial cells were flocculated and settled into the sediment in a short time (within 4 days). Cyanotoxins would be degraded quickly at the water-sediment interface (Chen et al. 2008).

### Recovering time of cyanobacteria

Phytoplankton community structure is related to the trophic state (Aizaki et al. 1986; Reynolds et al. 2000; Padisák et al. 2009), and change in nutrient concentration has a consequential impact on phytoplankton community. When available nutrients are depleted by flocculation (Sridhar et al. 1988; Aguilar et al. 2002), phytoplankton abundance, biomass, and chlorophyll *a* decline (Reynolds 2006). High light and nutrient availability favor the growth of *Microcystis* spp. and *Anabaena* spp. and reinforce their advantages for competition with other species (Havens et al. 1998; Nalewajko and Murphy 2001). Filamentous cyanobacteria such as *Cylindrospermopsis* spp. and *Limnothrix* spp. adapt to low light and low nutrient concentration (Isvánovics et al. 2000; Sinha et al. 2012). In our experiment, almost all cyanobacterial populations decreased rapidly in the first few days, and the trend of recovery depends on the dosage of RSBF. For example, in the RSBF_35_ treatment, phytoplankton did not recover until the 28th day, and the dominant species was replaced by species belonging to Chlorophyta. RSBF_25_ treatment affected cyanobacteria recovery on day 12 but cyanobacteria were no longer the only dominant group. *Microcystis* spp. and *Anabaena* spp. biomass in RSBF_15_ recovered close to the control group on day 12 but cyanobacteria once again dominated the phytoplankton community.

Cyanobacteria usually prefer alkaline environments and will lose their competition advantage in acid water (Kupriyanova et al. 2011). In the treatment enclosures with RSBF, pH values showed a gradient in the order: RSBF_15_ > RSBF_25_ > RSBF_35_. Recovery cyanobacterial biomass in the phytoplankton community corresponded to the gradient. pH value may control the recovering time of cyanobacteria.

### Effects of RSBF on water quality

In the present study, pH rapidly decreased to below four in the three treatments. This is attributed to Fe^3+^ hydrolysis, in which hydrogen ion increases with Fe^3+^ occurrence (Sunda and Huntsman 2003; Gálvez et al. 2008). Low pH tends to reduce the competitive advantage of cyanobacteria equipped with CO_2_ concentration means (Thoms et al. 2001). Iron has the ability to bind with phosphorus (Kleeberg et al. 2013). Our experiment showed that prepared flocculant with iron and chitosan can effectively reduce TP, TN, as well as chlorophyll *a*. The optimal condition for iron to bind with phosphate in flocculants with pH of between 5 and 7 (Cooke et al. 1993). Yuan et al. (2009) found that phosphorus sorption rates of LaCl_3_-modified clays were all higher than 90% in pH of 4–8, with a maximum of 97% at pH 5. Phosphate removal by kaolinite also depends on pH, with maximum phosphate removal occurring at low pH of 3–5. In our experiment, TP, SRP, and also TN decreased significantly within 4 days, when pH was about 4 (Fig. 2a).

Reservoirs in southern China usually experience an obvious thermal stratification in the dry season (Xiao et al. 2011). The settled flocs are difficult to be resuspended up into the water column, and nutrient-flocculated organic matters (including algae) at the bottom hardly supports cyanobacterial growth in the water column. Moreover, if RSBF is taken as a method to improve water quality in the long run, high concentration of red soil might contribute to isolate sediments from the overlying water like a thin blanket, and would inhibit the release of phosphorus by putting a ceiling on the sediment.

### Effects of RSBF on zooplankton

Toxicity of flocculation on zooplankton has been studied in laboratory and field experiments (Lewis et al. 2003; Seo et al. 2008). The EC_50_ of modified clay on the population growth of rotifer *B. calyciflorus* was calculated to be 0.15 g Phoslock® (van Oosterhout and Lürling 2013). Leachates of modified clay showed low toxicity to *Daphnia dubia* under 24.5 g clay/L, while Lanthanum addition was chronically toxic to *Daphnia magna* (Stauber 2000). Lürling and Tolman (2010) also found that low concentration of Phoslock modified clay has little effect on *Daphnia*. In our experiment, rotifers were rapidly reduced by RSBF to less than twp individuals per liter under the three RSBF treatments. RSBF_25_ and RSBF_35_ significantly reduced the abundance of cladocerans and copepods, but RSBF_15_ had less influence on the two zooplankton. High acidification can result in marked decrease in zooplankton biomass, abundance, and even species number (Geelen and Leuven 1986; Fischer et al. 2001; Vandysh 2002). Zooplankton species differ in their acid tolerance (Havens et al. 1993; Fischer et al. 2001). Rotifers have a very short life cycle, allowing them to be more sensitive to environmental factors than cladocerans and copepods (Kalff 2002).

Flocculants cause an abrupt perturbation on chemical and biological variables, which directly or indirectly influence zooplankton abundance and community structure (Urabe 1990; Seda and Devetter 2000; Gliwicz et al. 2010). For instance, zooplankton abundance will decrease with nutrient reduction that corresponds with food resource (Seda and Devetter 2000; Kalff 2002). Zooplankton prefers to graze edible-sized particles (< 30 μm) (Kalff 2002). In the control enclosures, phytoplankton was always dominated by indigestible cyanobacteria. After being treated with RSBF, the enclosures were almost devoid of phytoplankton, and although cyanobacteria and Chlorophyta somewhat recovered in the later period of the experiment, they were at a low biomass. Therefore, we speculate that food availability is changed after adding the flocculant, but it is difficult to be quantitatively evaluated, also see van Oosterhout and Lürling (2011) for *Daphnia*.

## Conclusions

The new modified flocculant with iron and chitosan can effectively remove cyanobacteria, especially for spherical cell species such as *Microcystis* spp. and *Anabaena* spp. Cyanobacteria will slowly recover according to the flocculant dosage. The red soil-based flocculant has a significant effect on zooplankton, but copepods and cladocera recovered fast. For its easy preparation, low impact on crustaceans and low cost, we suggest that the red soil-based flocculant is an effective material in removing cyanobacteria biomass for urgent management in tropical reservoirs for drinking water supply.
